# Effects of transcranial direct current stimulation on the rehabilitation of painful shoulder following a stroke: protocol for a randomized, controlled, double-blind, clinical trial

**DOI:** 10.1186/s13063-019-3266-y

**Published:** 2019-03-15

**Authors:** Janaina Andressa de Souza, João Carlos Ferrari Corrêa, Letizzia Dall’ Agnol, Filipe Ribeiro dos Santos, Márcia Rafaella Pereira Gomes, Fernanda Ishida Corrêa

**Affiliations:** 1 Rehabilitation Sciences, Universidade Nove de Julho (UNINOVE), Avenida Angélica, 1905-Apt161, São Paulo, SP Brasil; 2 Postgraduate program in Rehabilitation Sciences, Universidade Nove de Julho (UNINOVE), São Paulo, SP Brazil; 3 Undergraduate course in Physical Therapy, Universidade Nove de Julho (UNINOVE), São Paulo, Brazil

**Keywords:** Stroke, tDCS, Shoulder pain, Rehabilitation

## Abstract

**Background:**

Shoulder pain is reported to be one of the major challenges faced in the functional recovery of patients in rehabilitation following a stroke. In such cases, transcranial direct current stimulation (tDCS) has been used as an additional therapeutic tool for improvements in central and peripheral pain. The aim of the proposed study is to evaluate the effect of tDCS when combined with upper limb physical therapy on pain intensity and functional improvement in stroke survivors with shoulder pain in the hemiplegic limb.

**Methods:**

A randomized, placebo-controlled, double-blind, clinical trial is proposed. The volunteers will be randomly allocated to receive passive movement on the upper limb, which will be performed by the therapist for 20 min followed by either active tDCS or sham tDCS (current stimulation for 30 s) during simultaneous physical activity of the upper limb (“mini-bike”) for 20 min, totaling 40 min of intervention performed in 10 consecutive sessions. The anode electrode will be positioned over the primary motor cortex with a current of 2 mA and the cathode electrode will be positioned in the supraorbital region contralateral to the anode. The primary outcome will be shoulder pain intensity, which will be measured using the visual analog scale (VAS) on three occasions: 1) pre-intervention; 2) after 10 interventions (5 weekly sessions, for 2 weeks); and 3) 30 days after the end of the interventions. The secondary outcomes will be motor performance, upper limb function, and quality of life.

**Trial registration:**

Brazilian Registry of Clinical Trials, RBR-8F5MNY. Registered on June 2, 2017.

**Electronic supplementary material:**

The online version of this article (10.1186/s13063-019-3266-y) contains supplementary material, which is available to authorized users.

## Background

Stroke is the second most common cause of death and the third most common cause of disability [1]. This condition is therefore considered an important public health problem throughout the world due to the social and economic impacts stemming from motor, cognitive, and/or sensory impairments, which have a negative impact on the performance of activities of daily living [2].

Shoulder pain is a frequent complication of a stroke, with an incidence ranging from 34% to 85% [3]. It may start in the second week after a stroke, but its onset is much more frequent 2 to 4 months after the event [4]. Although some cases of poststroke shoulder pain disappear spontaneously throughout the rehabilitation process, 65% of stroke survivors report the persistence of this symptom, which may extend for 12 months or longer. According to the International Association for the Study of Pain (IASP) [5], 3 consecutive months with pain sensation may be considered the cut-off point for the division between acute and chronic pain, but 6 months is often used for purposes of research.

The pathophysiology of shoulder pain poststroke may have multifactorial causes. Authors suggest that it may be related to spasticity [6], musculoskeletal alteration [7], limitation of shoulder movements and subluxation, rotator cuff damage and changes in the general glenohumeral joint [8, 9], psychological factors and central changes [10, 11] due to thalamic and spinothalamic tract damage [12, 13].

The assessment and treatment of poststroke shoulder pain (PSSP) is largely based on the assumption that pain is due to biomechanical alterations within the shoulder joint after stroke. However, current treatment often provides limited pain relief, leading to a considerable number of patients with persistent pain. This suggests that PSSP may not be merely due to simple nociception from the shoulder joint [14]. Sensitization and possibly disinhibition seem to play a role in the chronic maintenance of this pain in the shoulder after stroke, and may explain why treatment aimed at reducing peripheral nociception is generally unsatisfactory. Unlike acute pain, chronic pain is no longer functional and may no longer be related to the initial cause. The underlying mechanisms for the development of chronic pain are not well understood. It is suggested that chronic pain is due to an imbalance of somatosensory inhibitory and excitatory modulation, favoring the facilitation of nociception [15].

The supraspinal somatosensory system may become sensitized, uninhibited, and/or functionally [16] or structurally reorganized [17] as a result of ongoing nociception. A reorganization of the primary sensory and motor cortex is reported to occur in a variety of chronic pain conditions [18–20]. Findings of cortical disinhibition in different chronic pain conditions provide further evidence of the interrelationship between motor and nociceptive systems and suggest a potential mechanism to explain the efficacy of stimulation of the primary motor cortex (M1) as a treatment modality for chronic pain. M1 is the main site selected for transcranial direct current stimulation (tDCS), which has significant analgesic effects in chronic pain conditions [21].

tDCS is defined as a noninvasive brain stimulation technique with neuromodulatory action that alters cortical activity and excitability [22, 23]. It is characterized by a continuous current of low intensity sufficient to decrease the depolarization threshold and, therefore, to facilitate the action potential in the neurons, as seen by researchers [24] that observing the effects of the anodic and cathodic tDCS when applied on the motor cortex with intensities of 0.2 and 1.0 mA for between 1 and 5 min. Among the observed effects, cortical excitability and amplitude of the motor evoked potential were increased by approximately 40% by the anodic tDCS, as well as a 30% reduction in motor evoked potential amplitude after cathodal stimulation. Thus, it is believed that the technique is able to modulate cortical excitability by altering the cellular potential of the membrane.

One study with fibromyalgia syndrome reports that increased excitability of this region of the brain enables the enhancement of motor control and, consequently, better pain control [25]. Another study reports that long periods of cortical stimulation can have lasting effects on brain function, with an increase in the intrinsic plasticity of the motor sensory system and the enhancement of the effects of other therapeutic tools [26]. In a study investigating the effect of tDCS administered over M1 on pain in patients with spinal cord injury, better effects were found when compared with sham tDCS [27]. In an investigation of the analgesic effects in different parts of the body of tDCS with anodal stimulation over M1 in patients with central poststroke pain, the authors found an improvement in sensory identification and analgesic effects [28]. No tDCS studies have been found in shoulder pain due to stroke.

There is evidence that pain relief in patients with chronic pain submitted to noninvasive brain stimulation may be related to the restoration of normal corticospinal and intracortical excitation induced by such stimulation [29, 30], as demonstrated in studies involving tDCS [31, 32]. According to some researchers, the effect of tDCS on chronic pain is due to the reinforcement of intracortical inhibition and a reduction in overfacilitation [33, 34].

The modulation of intracortical inhibition is also critical for fine motor control (the selective activation of muscles during motor tasks) [35] and is an important aspect of neuroplasticity associated with motor learning and rehabilitation [34, 36]. For example, experimental models involving conditioning tasks that result in a reduction in the stimulation of subliminal conditioning have been shown to impair subsequent motor learning tasks [37, 38], demonstrating the limitation of plasticity in the motor cortex [39]. Thus, it is possible that ongoing cortical disinhibition may be associated with impaired motor performance and motor learning in individuals with chronic pain.

Considering the relationship between pain and motor performance and the fact that long periods of cortical stimulation can have lasting effects on brain function, such as an increase in the intrinsic plasticity of the motor sensory system and the enhancement of the effectiveness of other therapeutic tools, it is believed that the combination of tDCS and physical therapy may lead to a reduction in pain. This combined therapy is expected to enhance the motor performance of patients with PSSP as well as improve both sleep quality and quality of life. Indeed, stimulation is effective at relieving a number of chronic pain conditions, especially in patients who are resistant to drug therapy [26, 40].

Therefore, the aim of the proposed study is to evaluate the effect of tDCS, when combined with upper limb physical therapy, on pain level and functional improvements in stroke patients with hemiplegic shoulder pain. As a secondary endpoint, we intend to correlate the analgesic effects of tDCS therapy and upper limb physical activity with the effects on motor performance and determine the influence of this therapeutic modality on sleep quality and quality of life.

## Methods

### Study design

A randomized, placebo-controlled, double-blind, longitudinal, clinical trial is proposed.

The primary outcome of this study will be the intensity of shoulder pain, which will be measured using the visual analog scale (VAS). The VAS will be administered on three occasions): 1) pre-intervention; 2), after 10 interventions; and 3) 30 days after the end of the interventions. The secondary outcomes are motor performance and quality of life.

Participants will be recruited from the physical therapy clinics of the University Nove de Julho, São Paulo, Brazil.

**Fig. 1:**
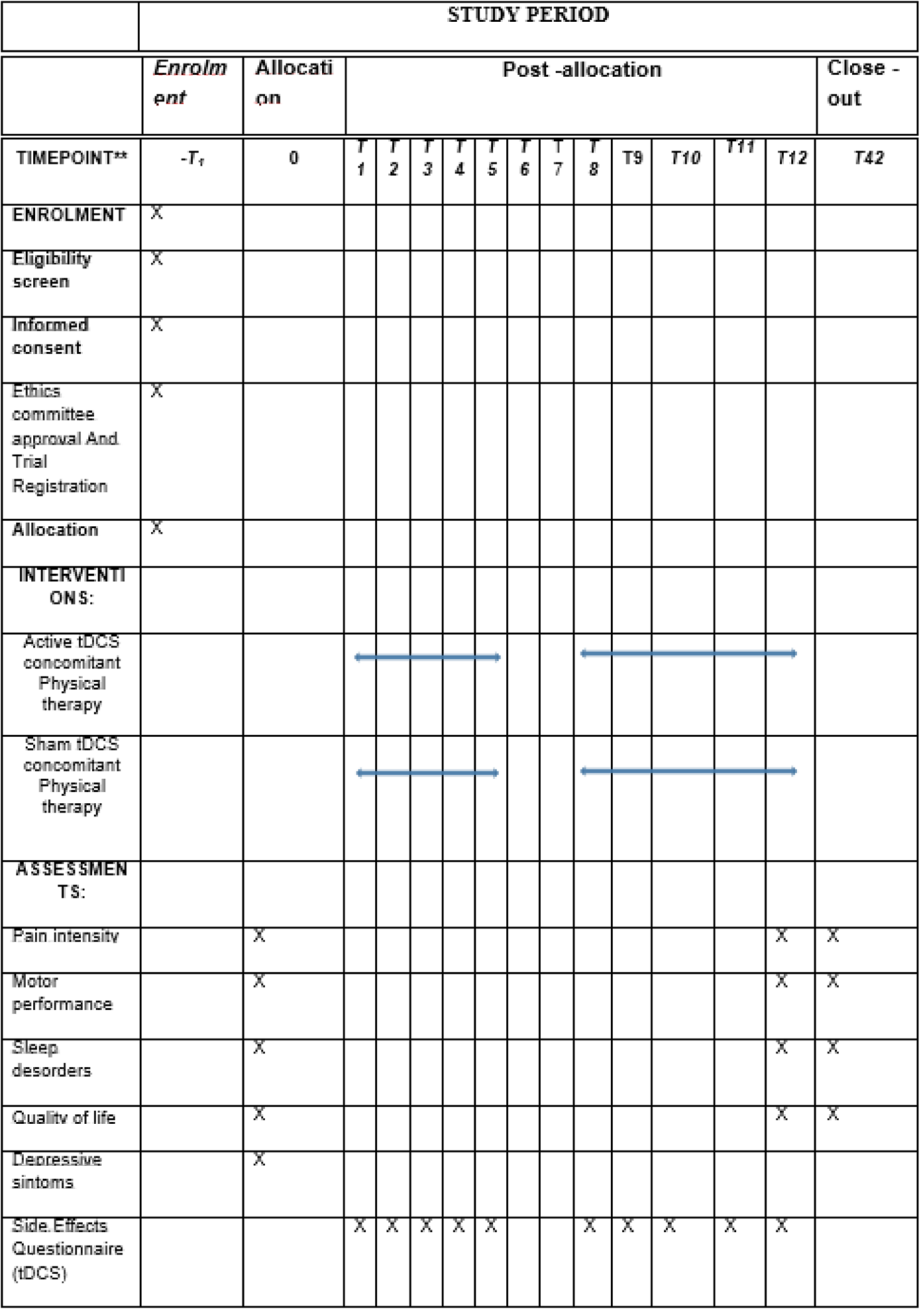
Diagram of the study. tDCS transcranial direct current stimulation

The SPIRIT checklist can be found as Additional file 1. The evolution of the study is shown in Fig. 1.

### Eligibility criteria

Individuals who meet the following inclusion criteria will be asked to participate in the study: 1) adults (> 18 years of age) with a diagnosis of chronic stroke [41] (there are few studies proving the safe administration of tDCS in the acute and subacute phases of a stroke, hence the choice of chronic stroke) and complaint of shoulder pain after stroke for at least 6 months (the ideal time for research on chronic pain, according to the IASP) [5]; and 2) able to understand commands (Mini-Mental State Examination score > 11) [42].

The exclusion criteria are: 1) contraindication for noninvasive brain stimulation (metal implants near application sites, history of seizures and/or epilepsy, pregnancy, and diagnosis of neoplasm); 2) muscular inelasticity (spasticity) > 3 on the affected upper limb, evaluated by the Modified Ashworth Scale [43]; 3) progressive neurological disease; 4) diagnosis of frozen shoulder; 5) severe sensory deficit (score > 2 on the National Institutes of Health (NIH) Stroke Scale) [44]; 6) diagnosis of acute coronary syndrome or severe heart problems (score > 3 on the New York Heart Association functional classification scale) [45]; 7) severe aphasia (score > 2 (evaluated in patient’s native tongue) indicated by NIH Stroke Scale) [44]; 8) suspicion or confirmation of recent upper limb fracture; 9) diagnosis of cancer and/or in therapy for palliative care; and 7) severe inattention (score > 2 indicated by NIH Stroke Scale) [44]. Concomitant care and interventions prohibited during the trial will be the recent use of botulinum toxin or phenol injection in the affected upper limb (in the previous 3 months) or a medical indication for use during the study period, the use of medications that can affect the evaluations (steroidal anti-inflammatory drugs), and participation in physical or homeopathic therapy during the study.

### Sample size

For the calculation of the sample size, the primary endpoint of the study (shoulder pain intensity at rest measured by VAS) was considered to be directly related to the intervention and has been widely used for this purpose. The calculation was performed considering a level of significance (alpha) of 0.05 and an 80% study power, considering a clinically important difference of ≥ 3 points on the VAS [46]. Based on the results of a pilot study involving 10 individuals with PSSP (five for the active group and five for the sham), the difference between the means was 3.0 in the group submitted to physical therapy, and active tDCS and 1.56 in the submitted group to physical therapy and sham ETCC, with a combined standard deviation of 1.38, the required sample size was 22 subjects (11 per group). Taking into account the possibility of dropouts, the sample was increased by 20% to ensure a sample size that enables us to demonstrate the effect of the intervention, leading to 13 individuals in each group (total of 26 participants). This calculation was performed using the G*Power software.

### Randomization

The allocation of the participants to the different groups will occur using a simple lottery procedure. Treatment codes (active or sham stimulation) will be placed in opaque envelopes [47, 48]. A researcher not involved in the evaluations or treatment will be responsible for the random allocation of the participants to two groups: 1) active tDCS combined with physical therapy for the upper limbs using a “mini-bike”; or 2) sham tDCS combined with physical therapy for the upper limbs using a “mini-bike”.

### Blinding

The NeuroConn DC-STIMULATOR PLUS device has settings that enable the selection of the active stimulation mode or sham mode by entering codes. A researcher not involved in the treatment or evaluations will program the equipment with the code to which the patient was allocated. The type of stimulation (active or sham) will not be perceptible by visual cues or the external functioning of the device. Therefore, neither the researcher who will place the equipment on the patient nor the patient will know which treatment he/she is receiving (a double-blind study).

### Data collection, management, and analysis

All evaluations will be performed prior at baseline (preintervention), after the 10 treatment sessions (postintervention), and 30 days after the end of the treatment sessions (follow-up).

### Pain intensity

Pain intensity will be measured using the VAS, which consists of a horizontal straight line measuring 10 cm, with 0 cm corresponding to the absence of pain and 10 cm corresponding to maximum pain [46, 49]. The interobserver and intraobserver reliability of this measure have been demonstrated using the intraclass correlation coefficient (ICC; 0.96 to 0.98) [49]. The assessments will be performed with the patient at rest (lying down) as well as during the active (performed by the subject) and passive movements (performed by the researcher) of the painful upper limb on three occasions: preintervention, postintervention, and 30-day follow-up. The intensity of the pain to be considered will be the pain at rest, without movement.

### Motor performance, upper limb function

The motor performance of the upper limb and its functioning will be evaluated on three occasions (preintervention, postintervention, and 30-day follow-up) using the measures below.

### Shoulder Pain and Disability Index (SPADI)

The SPADI is a questionnaire used to measure dysfunction and disability associated with shoulder pain [50]. The numerical version of the SPADI consists of 13 items, five of which address pain and eight of which address function. Each item is scored on a numerical scale from 0 to 10 points. For the final score, the points are converted into a percentage value (0 to 100%), with higher scores denoting worse shoulder dysfunction. The SPADI has been validated for use in the Brazilian population, with excellent test-retest reliability (ICC = 0.90 to 0.94) [50], and has been used in previous studies involving stroke survivors [51].

### Disabilities of the Arm, Shoulder and Hand (DASH)

The DASH is a questionnaire composed of 30 items developed to measure symptoms and disabilities caused by upper limb disorders in a heterogeneous population, the objective of which is to determine differences between groups of individuals through the comparison of the impact of upper limb disorder (mild, moderate, or severe disability) on physical functions and symptoms. The score ranges from 0 (absence of dysfunction) to 100 (severe dysfunction) [52]. DASH has been used in a previous study involving stroke survivors and demonstrated acceptable reliability (ICC = 0.97 to 0.99) [53].

### Fugl-Meyer Assessment of Motor Recovery after Stroke Scale

Motor function of the upper limbs will be analyzed using the Fugl-Meyer Assessment of Motor Recovery after Stroke Scale, which was designed specifically for the evaluation of patients with hemiparesis stemming from a stroke. The scale is divided into five domains: motor function, sensory function, balance, joint range of motion, and joint pain. The motor function domain includes the measurement of movement, coordination, and reflex activity of the shoulder, elbow, fist, hand, hip, and ankle, totaling 100 points (66 related to the upper half of the body and 34 related to the lower half) [54]. In the proposed study, only the score referring to the function of the upper half will be considered. The results of the scoring system enable the classification of the patient as having mild, moderate, or severe impairment. The Fugl-Meyer Assessment of Motor Recovery after Stroke has excellent reliability, validity, and sensitivity to change [55, 56]. This measure was translated into Brazilian Portuguese based on the original version published in 1975 [54], demonstrating high interobserver and intraobserver reliability for the total scale (ICC = 0.99 and 0.98, respectively) as well as the upper extremity motor function subscale (ICC = 0.99 and 0.98, respectively) [57].

### Shoulder range of motion

A goniometer, which is a device consisting of two plastic rods (one fixed and one movable) and a compass to allow determination of joint angles [58], will be used to measure shoulder angles in the range of external rotation, abduction, and flexion during passive movements performed by the researcher and active movements performed by the volunteer.

### Grip strength

For this evaluation, the volunteer will be seated with the upper limbs supported on the arm rests of the chair and the elbows at 90 degrees. Grip strength will be measured in both hands using the Jamar® dynamometer (Enterprises Inc., Irvington, New York, USA) with the handle in the second position, which results in greater grip strength [59]. The American Society of Hand Therapists also recommends the use of position II as a standard in clinical practice and research during grip strength tests involving the Jamar dynamometer. The reliability of this device is high (ICC > 0.87) [60].

### Quality of life

Quality of life will be measured using the Stroke-Specific Quality-of-Life Scale (SSQOL), which is a standardized, validated scale that specifically addresses the quality of life of stroke survivors [61]. The scale is composed of 49 items distributed among 12 domains. Each item is scored from 1 to 5 points. The total ranges from 49 to 245, with higher scores denoting better quality of life. The SSQOL has a reliability coefficient of 0.92 [61] and has been used in studies with good clinical results [62].

### Determination of potential confounding factors

#### Depressive symptoms

Depressive symptoms will be evaluated and graded with regard to severity using the Beck Depression Inventory (BDI), which is a self-administered questionnaire composed of 21 items. Each item is scored from 0 to 3 points. The total ranges from 0 to 63 points and is interpreted as follows: 0 to 10 indicates the absence of depression; 11 to 18 = mild depression; 19 to 29 = moderate depression; and 30 to 63 = severe depression [63, 64]. The BDI score will be determined on three occasions (preintervention, postintervention, and 30-day follow-up) and will be used as a covariant to determine whether motor recovery is independent of possible mood-related effects [65]. The reliability of the BDI is 0.89 [64] and this measure has been used in studies that have shown good clinical results [62].

#### Sleep disorders

Sleep-related disorders will be analyzed using the Pittsburgh Sleep Quality Index (PSQI). This questionnaire is composed of 24 items, 19 of which are self-administered and subdivided into seven domains: 1) subjective sleep quality; 2) sleep latency; 3) duration of sleep; 4) habitual effectiveness of sleep; 5) sleep disorders; 6) use of sleeping pills; and 7) daytime dysfunctions. Five additional questions are answered by the person who shares the same bedroom as the individual participating in the evaluation. The sum of the seven domains ranges from 0 to 21 points, with higher scores denoting poorer sleep quality. A score greater than 5 indicates major difficulties in at least 2 components or moderate difficulties in more than 3 components. The PSQI will be determined at the preintervention evaluation and 30-day follow-up. The reliability of this measure is 0.82 (general reliability coefficient) [66, 67].

#### Use of medications

The use and amount of medications, such as analgesics, anti-inflammatory agents, antidepressants, and anticonvulsants, will be measured with the aid of a questionnaire designed by the authors of the study, which will contain the complete data of each patient.

#### Intervention

All patients will receive passive manipulations, such as massage, stretching, and passive joint movements, which will be administered by a therapist on the painful upper limb for 20 min. Next, tDCS will be administered over the cerebral cortex and the patient will receive stimulation for 20 min while performing physical activity with the upper limbs (“mini-bike”). The total physiotherapeutic treatment will be 40 min. Active or sham tDCS and physical activity will be performed at a frequency of five sessions per week for 2 weeks, totaling 10 sessions [68].

#### Transcranial direct current stimulation (tDCS)

Stimulation will be performed using the NeuroConn DC-STIMULATOR PLUS and administered over the primary motor cortex (M1) of the damaged hemisphere. Unbalanced bipolar anodal stimulation will be administered with the anode positioned over either C3 (left hemisphere) or C4 (right hemisphere), following the 10/20 electroencephalogram system. The cathode will be positioned in the supraorbital region contralateral to the anode [69]. The cathode will measure 35 cm^2^ (5 × 7 cm) and the anode will measure 25 cm^2^ (5 × 5 cm). Both electrodes will be enveloped in sponge soaked in saline solution. During active tDCS, the intensity of the current will be 2 mA, administered for 20 min.

#### Sham transcranial direct current stimulation

Sham stimulation will be performed with the device in the sham setting. The montage will be the same as that in the active stimulation group, but the device will only be activated for 60 s, with a gradual increase in current during the first 30 s (ramp up) to give the volunteer the sensation of stimulation, followed by a gradual decrease over the subsequent 30 s (ramp down). Sham tDCS will be administered by a therapist who will not participate in the evaluations.

#### Determination of potential side effects

Possible adverse effects stemming from noninvasive brain stimulation will be determined using the TDCS Side Effects Questionnaire (a version translated into Portuguese) after each session and immediately after the intervention protocol [70].

#### Physical therapy

Physical activity will consist of active movements of the upper limbs using a “mini-bike” (Acte Sports, model E5). For this, the patient will be positioned in a chair with a 90° backrest and feet resting on the floor. The mini-bike will be positioned on a table, which will allow free arm movements. The patient will perform active movement or active-assisted movements (if the patient cannot hold onto the handle, the therapist will assist by securing the patient’s hand on the handle) [71]. This activity will be performed for 20 min concomitantly with tDCS.

#### Data analysis

Descriptive data and characteristics of the patients (gender, age, initial score on the VAS, ischemic or hemorrhagic stroke, right/left hemisphere lesion, time with pain, injury time, Fugl-Meyer upper limb score, BDI, use of controlled medications, and associated comorbidities) will be represented by mean and standard deviation or median and interquartile range. The normality of the data from the VAS, DASH, Fugl-Meyer, SSQOL, range of motion passive shoulder, and hand grip strength will be analyzed by the Shapiro-Wilk test.

If the normality hypothesis is accepted, the analysis of variance (ANOVA) followed by the Bonferroni test will be used, when necessary, to perform the comparison analysis between groups. If the normality hypothesis is rejected, the Kruskal-Wallis test will be used. The Mann-Whitney test will be used to compare the results of the study treatment periods (pre- and post-treatment and 30-day follow-up). Pearson or Spearman correlation coefficients will be calculated to determine the strength of correlations between pain and secondary outcomes (SPADI, Fugl-Meyer, SSQOL, passive shoulder range of motion and grip strength). A value of *p* < 0.05 will be considered indicative of statistical significance. All analyses will be processed using the SPSS program (IBM SPSS Statistics for Windows, Version 22.0, released in 2013, IBM Corp., Armonk, NY, USA).

### Trial status

At the time of manuscript submission we were recruiting patients. The study in question is expected to be completed in November 2018.

## Additional file

Additional file 1: SPIRIT 2013 checklist: recommended items to address in a clinical trial protocol and related documents. (DOC 122 kb)

## Abbreviations

BDI: Beck Depression Inventory
C3: Left hemisphere
C4: Right hemisphere
DASH: Disabilities of the Arm, Shoulder and Hand
IASP: International Association for the Study of Pain
M1: Primary motor cortex
NIH: National Institutes of Health
PSQI: Pittsburgh Sleep Quality Index
PSSP: Poststroke shoulder pain
SPADI: Shoulder Pain and Disability Index
SSQOL: Stroke-Specific Quality-of-Life Scale
tDCS: Transcranial direct current stimulation
VAS: Visual analog scale

## References

[1] Johnson W, Onuma O, Owolabi M, Sachdev S (2016). Stroke: a global response is needed. Bull World Health Organ.

[2] Park S, Park JY (2016). Grip strength in post-stroke hemiplegia. J PhysTher Sci.

[3] Vuagnat H, Chantraine A (2003). Shoulder pain in hemiplegia revisited: contribution of functional electrical stimulation and other therapies. J Rehabil Med.

[4] Poduri KR (1993). Shoulder pain in stroke patients and its effects on rehabilitation. J Stroke Cerebrovasc Dis.

[5] International Association for the Study of Pain (IASP). Part III: pain terms: a current list with definitions and notes on usage. Classification of chronic pain. 2012; Available at: https://www.iasp-pain.org/PublicationsNews/Content.aspx?ItemNumber=1673. Accessed 13 Mar 2019.

[6] Stevenson VL (2010). Rehabilitation in practice: spasticity management. Clin Rehabil.

[7] Gardner MJ, Ong BC, Liporace F, Koval KJ (2002). Orthopedic issues after cerebrovascular accident. Am J Orthop.

[8] Widar M, Samuelsson L, Karlsson-Tivenius S, Ahlström G (2002). Long-term pain conditions after a stroke. J Rehabil Med.

[9] Lundström E, Smits A, Terént A, Borg J (2009). Risk factors for stroke-related pain 1 year after first-ever stroke. Eur J Neurol.

[10] Klint H, Finnerup NB, Overvad K, Andersen G, Jensen TS (2011). Pain following stroke: a population-based follow-up study. PLoS One.

[11] Zeilig G, Rivel M, Doron D, Defrin R (2016). Does hemiplegic shoulder pain share clinical and sensory characteristics with central neuropathic pain? A comparative study. Eur J Phys Rehabil Med.

[12] Demasles S, Peyron R, Garcia Larrea L, Laurent B (2008). Central post-stroke pain. Rev Neurol (Paris).

[13] Henry JL, Lalloo C, Yashpal K (2008). Central poststroke pain: an abstruse outcome. Pain Res Manag.

[14] Roosink M, Renzenbrink GJ, Geurts ACH, IJzerman MJ (2012). Towards a mechanism-based view on the post-stroke shoulder pain: theoretical considerations and clinical implications. NeuroRehabilitation.

[15] Curatolo M, Arendt-Nielsen L, Petersen-Felix S (2006). Central hypersensitivity in chronic pain: mechanisms and clinical implications. Phys Med Rehabil Clin N Am.

[16] Willoch F, Schindler F, Wester HJ, Empl M, Straube A, Schwaiger M (2004). Central poststroke pain and reduced opioid receptor binding within pain processing circuitries: a [11C]diprenorphine PET study. Pain..

[17] Karl A, Birbaumer N, Lutzenberger W (2001). Reorganization of motor and somatosensory cortex in upper extremity amputees with phantom limb pain. J Neurosci.

[18] Roe Y, Soberg HL, Bautz-Holter E, Ostensjo S (2013). A systematic review of measures of shoulder pain and functioning using the international classification of functioning, disability and health (ICF). BMC Musculoskelet Disord.

[19] Adey-Wakeling Z, Arima H, Crotty M, Leyden J, Kleinig T, Anderson CS (2015). Incidence and associations of hemiplegic shoulder pain poststroke: prospective population-based study. Arch Phys Med Rehab.

[20] Latremoliere A, Woolf CJ (2009). Central sensitization: a generator of pain hypersensitivity by central neural plasticity. J Pain.

[21] Lefaucheur JP, André-Obadia N, Antal A, Ayache SS, Baeken C, Benninger DH (2014). Evidence-based guidelines on the therapeutic use of repetitive transcranial magnetic stimulation (rTMS). Clin Neurophysiol.

[22] Das S, Holland P, Frens MA, Donchin O (2016). Impact of transcranial direct current stimulation (tDCS) on neuronal functions. Front Neurosci.

[23] Nitsche MA, Cohen LG, Wassermann EM, Priori A, Lang N, Antal A (2008). Transcranial direct current stimulation: state of the art 2008. Brain Stimul..

[24] Nitsche MA, Paulus W (2000). Excitability changes induced in the human motor cortex by weak transcranial direct current stimulation. J Physiol.

[25] Busch AJ, Barber KA, Overend TJ, Peloso PM, Schachter CL. Exercise for treating fibromyalgia syndrome. Cochrane Database Syst Rev. 2007;17;(4):CD003786. 10.1002/14651858.CD003786.pub2 PMC12832165 17943797

[26] Shafi MM, Westover MB, Fox MD, Pascual-Leone A (2012). Exploration and modulation of brain network interactions with noninvasive brain stimulation in combination with neuroimaging. Eur J Neurosci.

[27] Ngernyam N, Jensen MP, Arayawichanon P, Auvichayapat N, Tiamkao S, Janjarasjitt S (2015). The effects of transcranial direct current stimulation in patients with neuropathic pain from spinal cord injury. Clin Neurophysiol.

[28] Bae SH, Kim GD, Kim KY (2014). Analgesic effect of transcranial direct current stimulation on central post-stroke pain. Tohoku J Exp Med.

[29] Lefaucheur JP, Drouot X, Ménard-Lefaucheur I, Keravel Y, Nguyen JP (2006). Motor cortex rTMS restores defective intracortical inhibition in chronic neuropathic pain. Neurology..

[30] Hosomi K, Kishima H, Oshino S, Hirata M, Tani N, Maruo T (2013). Cortical excitability changes after high-frequency repetitive transcranial magnetic stimulation for central post stroke pain. Pain..

[31] Fregni F, Gimenes R, Valle AC, Ferreira MJ, Rocha RR, Natalle L, et al. A randomized, sham-controlled, proof of principle study of transcanial direct current stimulation for the treatment of pain in fibromyalgia. Arthritis Rheum. 2006b;54(12):3988–98. 10.1002/art.22195 17133529

[32] Zimerman M, Heise KF, Hoppe J, Cohen LG, Gerloff C, Hummel FC (2012). Modulation of training by single-session transcranial direct current stimulation to the intact motor cortex enhances motor skill acquisition of the paretic hand. Stroke..

[33] Antal A, Terney D, Kühnl S, Paulus W (2010). Anodal transcranial direct current stimulation of the motor cortex ameliorates chronic pain and reduces short intracortical inhibition. J Pain Symptom Manag.

[34] Ljubisavljevic M (2006). Transcranial magnetic stimulation and the motor learning-associated cortical plasticity. Exp Brain Res.

[35] Liepert J, Classen J, Cohen LG, Hallett M (1998). Task-dependent changes of intracortical inhibition. Exp Brain Res.

[36] Smyth C, Summers JJ, Garry MI (2010). Differences in motor learning success are associated with differences in M1 excitability. Hum Mov Sci.

[37] Sriraman A, Oishi T, Madhavan S (2014). Timing-dependent priming effects of tDCS on ankle motor skill learning. Brain Res.

[38] Kuo M-F, Unger M, Liebetanz D, Lang N, Tergau F, Paulus W (2008). Limited impact of homeostatic plasticity on motor learning in humans. Neuropsychologia..

[39] Bienenstock EL, Cooper LN, Munro PW (1982). Theory for the development of neuron selectivity: orientation specificity and binocular interaction in visual cortex. J Neurosci.

[40] Tsubokawa T, KatayamaY YT, Hirayama T, Koyama S (1991). Chronic motor cortex stimulation for the treatment of central pain. Acta Neurochir Suppl (Wien).

[41] Bernhardt J, Hayward KS, Kwakkel G, Ward NS, Wolf SL, Borschmann K (2017). Agreed definitions and a shared vision for new standards in stroke recovery research: The Stroke Recovery and Rehabilitation Roundtable taskforce. Int J Stroke.

[42] Bertolucci PHF, Brucki SMD, Campacci SR, Juliano Y (1994). O Mini-Exame do Estado Mental em uma população geral: Impacto da Escolaridade. NeuroPsiquiatr..

[43] Bohannon RW, Smith MB (1987). Interrater reliability of a modified Ashworth scale of muscle spasticity. Phys Ther.

[44] Adams HP, Davis PH, Leira EC, Chang KC, Bendixen BH, Clarke WR (1999). Baseline NIH Stroke Scale score strongly predicts outcome after stroke: a report of the Trial of Org 10172 in Acute Stroke treatment (TOAST). Neurology..

[45] Bennett JA, Riegel B, Bittner V, Nichols J (2002). Validity and reliability of the NYHA classes for measuring research outcomes in patients with cardiac disease. Heart Lung.

[46] Jensen MP, Chen C, Brugger AM (2003). Interpretation of visual analog scale ratings and change scores: a reanalysis of two clinical trials of post operative pain. J Pain.

[47] Huang YC, Leong CP, Tso HH, Chen MJ, Liaw MY, Hsieh HC (2018). The long-term effects of hyaluronic acid on hemiplegic shoulder pain and injury in stroke patients: a randomized controlled study. Medicine (Baltimore).

[48] Karasu AU, Batur EB, Karatas GK (2018). Effectiveness of Wii-based rehabilitation in stroke: a randomized controlled study. J Rehabil Med.

[49] Bijur PE, Silver W, Gallagher EJ (2001). Reliability of the visual analog scale for measurement of acute pain. Acad Emerg Med.

[50] Martins J, Napoles BV, Hoffman CB, Oliveira AS (2010). The Brazilian version of Shoulder Pain and Disability Index: translation, cultural adaptation and reliability. Rev Bras Fisioter..

[51] Pandian JD, Kaur P, Arora R, Vishwambaran DK, Toor G, Mathangi S (2013). Shoulder taping reduces injury and pain in stroke patients: randomized controlled trial. Neurology..

[52] Orfale AG, Araujo PM, Ferraz MB, Natour J (2005). Translation into Brazilian Portuguese, cultural adaptation and evaluation of the reliability of the Disabilities of the Arm, Shoulder and Hand Questionnaire. Braz J Med Biol Res.

[53] Dalton E, Lannin NA, Laver K, Ross L, Ashford S, McCluskey A (2016). Validity, reliability and ease of use of the disabilities of arm, shoulder and hand questionnaire in adults following stroke. Disabil Rehabil.

[54] Fugl-Meyer AR, Jaasko L, Leyman I, Olsson S, Steglind S (1975). The post-stroke hemiplegic patient. Scand J Rehabil Med.

[55] Gladstone DJ, Danells CJ, Black SE (2002). The Fugl-Meyer assessment of motor recovery after stroke: a critical review of its measurement properties. Neurorehabil Neural Repair.

[56] Woodbury ML, Velozo CA, Richards LG, Duncan PW, Studenski S, Lai SM (2007). Dimensionality and construct validity of the Fugl-Meyer assessment of the upper extremity. Arch Phys Med Rehabil.

[57] Maki T, Quagliato EMAB, Cacho EWA, Paz LPS, Nascimento NH, Inoue MMEA (2006). Estudo da confiabilidade da aplicação da escala de Fugl-Meyer no Brasil. Ver Bras Fisioter.

[58] Marques AP (2003). Manual de Goniometria. 2 ed.

[59] Figueiredo I, Sampaio RF, Mancini MC, Silva FCM, Souza MAP (2007). Teste de força de preensão utilizando o dinamômetro Jamar. Acta Fisiátrica.

[60] MacDermid JC, Kramer JF, Woodbury MG, McFarlane RM, Roth JH (1994). Interrater reliability of pinch and grip strength measurements in patients with cumulative trauma disorders. J Hand Ther.

[61] Lima RCM, Teixeira-Salmela LF, Magalhães LC, Gomes NM (2008). Propriedades psicométricas da versão brasileira da escala de qualidade de vida específica para acidente vascular encefálico: aplicação do modelo Rasch. Rev Bras Fisioter.

[62] An TG, Kim SH, Kim KU (2017). Effect of transcranial direct current stimulation of stroke patients on depression and quality of life. J Phys Ther Sci.

[63] Gorenstein C, Andrade L (1998). Inventário de depressão de Beck: propriedades psicométricas da versão em português. Rev Psiquiatr Clín.

[64] Gomes-Oliveira MH, Gorenstein C, Lotufo Neto F, Andrade LH, Wang YP (2012). Validation of the Brazilian Portuguese version of the Beck Depression Inventory-II in a community sample. Rev Bras Psiquiatr São Paulo.

[65] Kroenke K, Spitzer RL, Williams JB (2001). The PHQ-9: validity of a brief depression severity measure. J Gen Intern Med.

[66] Buysse DJ, Reynolds CF, Monk TH, Berman SR, Kupfer DJ (1989). The Pittsburgh Sleep Quality Index: a new instrument for psychiatric practice and research. Psychiatry Res.

[67] Bertolazi AN, Fagondes SC, Hoff LS, Dartora EG, Miozzo IC, de Barba ME (2011). Validation of the Brazilian Portuguese version of the Pittsburgh Sleep Quality Index. Sleep Med.

[68] Fregni F, Boggio PS, Lima MC, Ferreira MJ, Wagner T, Rigonatti SP (2006). A sham-controlled, phase II trial of transcranial direct current stimulation for the treatment of central pain in traumatic spinal cord injury. Pain.

[69] Lefaucheur JP, Antal A, Ahdab R, Ciampi de Andrade D, Fregni F, Khedr EM (2008). The use of repetitive transcranial magnetic stimulation (rTMS) and transcranial direct current stimulation (tDCS) to relieve pain. Brain Stimul.

[70] Xu J, Fregni F, Brody AL, Rahman AS (2013). Transcranial direct current stimulation reduces negative affect but not cigarette craving in overnight abstinent smokers. Front Psychiatry.

[71] Haddad S (1997). Ergometria de membros superiores. Um método importante na avaliação cardiocirculatória ao exercício. Arq Bras Cardiol.

